# SARS-CoV-2 envelope protein mitochondrial localization reveals host metabolic disruption

**DOI:** 10.1016/j.jbc.2026.111419

**Published:** 2026-04-01

**Authors:** Emily A. David, Elijah J. Bass, Hannah M. Woods, Daniel Stephenson, Kellyann Román-Cruz, Charles E. Chalfant, Robert V. Stahelin

**Affiliations:** 1 Borch Department of Medicinal Chemistry and Molecular Pharmacology, Purdue College of Pharmacy, Purdue Institute of Inflammation, Immunology, and Infectious Disease, Purdue University, West Lafayette, Indiana, USA; 2 Division of Hematology and Oncology, University of Virginia-School of Medicine, Charlottesville, Virginia, USA; 3 Research Service, Richmond Veterans Administration Medical Center, Richmond, Virginia, USA

**Keywords:** cellular localization, envelope protein, metabolism, membrane potential, mitochondria, reactive oxygen species, SARS-CoV-2

## Abstract

Severe acute respiratory syndrome coronavirus 2 is an enveloped virus that encodes four structural proteins, including the small transmembrane envelope (E) protein. While E is known to function in viral assembly and egress, it contributes to host cell dysfunction and disease severity. We demonstrate that severe acute respiratory syndrome coronavirus 2 E localizes to host cell mitochondria and alters mitochondrial structure, metabolism, and redox homeostasis. Using fluorescence microscopy, we observed that E forms tubular cytoplasmic structures that colocalize with mitochondria and ceramide-rich domains. Lipidomic analysis revealed that E expression leads to reductions in cardiolipin, phosphatidylcholine, and lysophospholipids. Mitochondrial membrane potential was decreased in E-expressing cells, consistent with disrupted electron transport chain activity, which was further supported by mitochondria stress testing *via* Seahorse. Despite increased mitochondrial reactive oxygen species, E did not trigger apoptosis, suggesting containment of oxidative stress within the organelle. Metabolomic profiling revealed decreased levels of key glycolytic and tricarboxylic acid cycle intermediates, along with altered GSH and sulfur metabolism. Notably, glutamine levels increased, potentially to compensate for reduced 2-oxoglutarate. Together, these findings suggest that E protein localizes to the mitochondria, perturbs lipid and metabolic homeostasis, and promotes reactive oxygen species retention without inducing cell death. This mitochondrial dysfunction may support a shift toward aerobic glycolysis, facilitating viral replication. Our study highlights an underappreciated role for E in modulating host metabolism.

Severe acute respiratory syndrome coronavirus 2 (SARS-CoV-2) is an enveloped virus with a positive-sense single-stranded RNA genome. This viral genome encodes four structural proteins that assemble to form the mature virion and biosafety level 2–compatible virus-like particles (VLPs) (1). These four structural proteins are membrane (M), envelope (E), nucleoprotein (N), and spike (S) (1). S protein binds to angiotensin-converting enzyme 2 on the cell surface to facilitate receptor-mediated endocytosis (2). Upon entry into the host, endosomal acidification facilitates the release of the RNA genome and subsequent translation and replication of the RNA genome (2). M is a transmembrane dimer that forms a protein matrix in the endoplasmic reticulum (ER)–Golgi intermediate compartment (ERGIC), whereas N packages the RNA genome (3, 4). Once N oligomerizes to form a complex with the RNA genome, called a nucleocapsid, the nucleocapsid traffics to the site of assembly, where M oligomers reside in the ERGIC (4, 5). The E protein is known to localize to the ER (6) and ERGIC membranes with M (7, 8). Both E and M are required for viral budding, working together to induce membrane curvature and the budding off of the ERGIC membrane (7, 8, 9, 10, 11). Upon completion of assembly and budding, the ERGIC-derived virions are then trafficked to the plasma membrane *via* lysosomes for subsequent release into the extracellular space (4, 12).

The E protein is a small (∼8.5 kDa) structural protein known to influence membrane curvature (13, 14, 15) and functions as a viroporin, disrupting cellular homeostasis through multiple pathways (16, 17). E has an important function in the pathogenicity of both SARS-CoV-2 infection and the inflammatory cascades that characterize severe coronavirus disease 2019 (COVID-19) infection (9, 18, 19, 20), as well as its predecessor, SARS-CoV (7, 21, 22). Among the four structural proteins, E has the highest degree of sequence conservation among a variety of coronaviruses and is 94.74% identical to its precursor SARS-CoV (9, 23).

E plays a vital role in the overall morphology of the viral envelope, whereas being the least abundant structural protein in both the native virus and the VLP system (1, 7, 24, 25). E consists of 75 amino acids, which include an N-terminal domain, transmembrane domain, and C-terminal domain (CTD). The CTD of E contains a PDZ (postsynaptic density protein/disc large tumor suppressor/zonula occludens-1 protein) domain–binding motif, which enables interactions with a variety of host proteins, such as syntenin1, thus initiating the cascade that leads to hypercytokinemia and cell death (16, 26). E interacts with the host protein associated with LIN7 1, MAGUK P55 family member, PALS1 (protein associated with LIN7 1; MAGUK p55 family member), a tight junction–associated protein that maintains the structural integrity of epithelial tissues within the lungs (9, 27, 28, 29). Disruption of the tight junction leads to the development of acute respiratory distress syndrome, which results from the buildup of fluid in the lungs when the epithelial cells become more permeable as a potentially fatal complication of a hyperinflammatory response (16, 20, 30, 31). The specific cause of acute respiratory distress syndrome was linked to the activity of the SARS-CoV E protein (9, 20). The PDZ domain–binding motif and the amyloidization motif, which facilitates oligomerization of the E protein into viroporins (32, 33, 34), have been identified as critical for the release of VLPs (35).

The oligomerization of E results in functional pentamers that organize into viroporins (36), an ion channel that allows the passage of potassium, chloride, and calcium ions (15, 37). The E CTD also retains localization sequences for the ER and Golgi membranes (38). SARS-CoV-2 infection causes the alkalinization of the ERGIC, where E's role in this flux in pH was revealed *via* mutagenesis (8). Lysosomal acidification was found to be correlated with the increased expression of E (35). The resulting changes in pH and charge within host organelles cause cell stress that induces a cytokine-mediated inflammatory response (15, 16, 27). M and E have been shown to play a critical role in calcium flux at ER and mitochondrial contact points, with E acting as a bridge between the two organelles (39).

There is a complex relationship between host cell mitochondrial dysfunction and the dsRNA replicative intermediate of SARS-CoV-2. dsRNA has been shown to localize to the mitochondria and alter the morphology and homeostasis of the mitochondrial membranes (40). SARS-CoV-2 was used in concert with dsRNA staining, so the direct relationship between SARS-CoV-2 virions and mitochondrial dysfunction remained unclear (40). The same study revealed that infection with SARS-CoV-2 causes mitochondrial depolarization, but dysfunctional mitochondria were no longer broken down in autophagosomes, a process known as mitophagy (40). The joint expression of E and ORF3a, another SARS-CoV-2 viroporin, showed an increase in mitochondrial calcium, the production of reactive oxygen species (ROS), subsequent release of mitochondrial DNA into the cytosol, and activation of nucleotide-binding domain, leucine-rich–containing family, pyrin domain–containing-3-inflammasome (17).

In this study, we further examined E subcellular localization and its ability to alter host metabolism. Previous work has shown that host ceramide (Cer) levels increase following SARS-CoV-2 infection (41). Cer structurally generates negative curvature in membranes, and given Cer’s membrane-altering properties (42, 43), we hypothesized that E might use Cer as a platform to localize for assembly and membrane budding. Here, we use an array of cellular imaging to visualize E protein localization and lipidomics and metabolomics to analyze how E alters host cell metabolism. Our work highlights the localization of the E protein to the mitochondria and its effects on metabolic and lipid changes in the mitochondrial environment. Altogether, these findings can be used to better understand how E induces changes to host cell biology, independently of other SARS-CoV-2 proteins, thus developing into a potential target for future therapeutics.

## Results

### SARS-CoV-2 E colocalizes with the mitochondria

**Figure 1:**
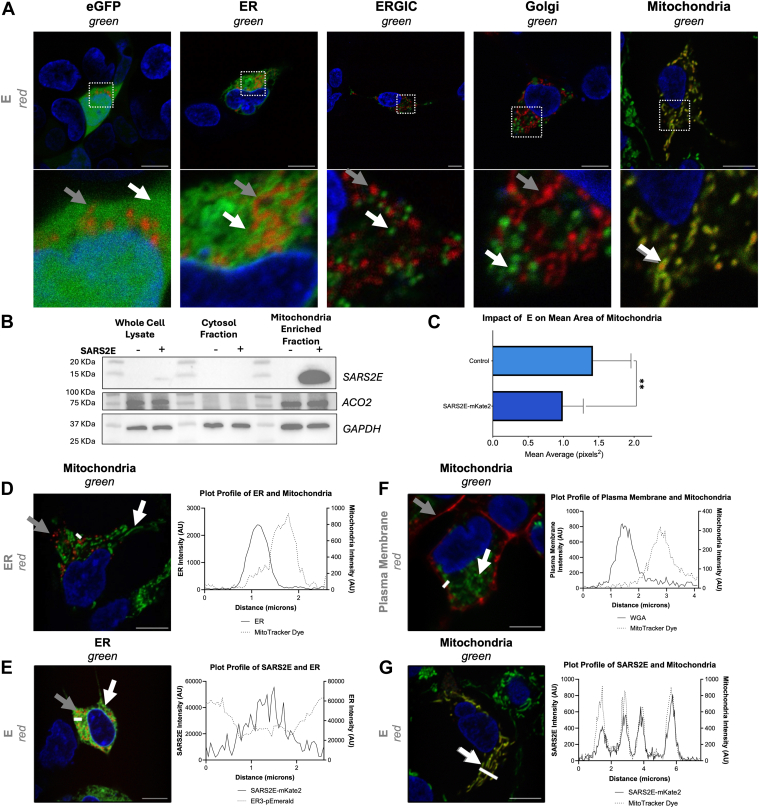
**Localization of SARS2E to mitochondrial membranes**. *A*, HEK293 cells transfected with SARS2E-mKate2 (*redchannel* and *gray arrow*) and cellular markers including EGFP (*green* and *white arrow*) (n = 26), ER-mEmerald3 (*green* and *white arrow*) (n = 26), ERGIC53-mEmerald3 (*green* and *white arrow*) (n = 29), Golgi7-mEmerald (*green* and *white arrow*) (n = 35), or MitoTracker Green (*green* and *white arrow*) (n = 19), (*left* to *right*) were analyzed by confocal microscopy 24 h post-transfection. The cells were counterstained with Hoechst 33342 (*blue*). The *lower panel* is a zoomed-in version of each cell as highlighted by the *white box* in the *upper panel* images. Please note that the GFP image shown in this panel is the same GFP image shown in Figure S1*B* for comparison purposes across the two figures. *B*, immunoblotting of HEK293T transfected and selected for SARS2E or mock, mitochondrial fractions isolated (n = 3), and separated by a gradient 4% to 20% SDS-PAGE. *C*, mitochondria morphology comparisons were made using the MitoTracker Green signal, averaging the mitochondria's mean area and Welch’s *t* test following ROUT removal of outliers to Q = 1%. *D*–*G*, images and plot profiles of HEK293 cells transfected with ER3-mCherry (*red channel* and *gray arrow*) and stained with MitoTracker Green (*green channel* and *white arrow*) in *D*. HEK293 cells transfected with ER3-mEmerald (*green channel* and *white arrow*) and SARS2-E-mKate2 (*red channel and gray arrow*) in *E*. HEK293 cells mock transfected with MitoTracker Green (*green channel* and *white arrow*) and stained with WGA plasma membrane stain (*red channel* and *gray arrow*) in *F*. HEK293 cells transfected with SARS2-E-mKate2 (*red channel* and *gray arrow*) and stained with MitoTracker Green (*green channel* and *white arrow*) in *G*. *D*–*G*, the cells were counterstained with Hoechst 33342 (*blue*) 24 h post-transfection, and the plot profile for the intensity of signal over the representative distance was determined. The scale bar represents 10 μm in all images. EGFP, enhanced GFP; ER, endoplasmic reticulum; ERGIC, ER–Golgi intermediate compartment; HEK293, human embryonic kidney 293 cell line; SARS2E, SARS-CoV-2 E protein (envelope protein of severe acute respiratory syndrome coronavirus 2); WGA, wheat germ agglutinin.

When E tagged with mKate2 (SARS2E-mKate2) was expressed in human embryonic kidney 293 (HEK293) cells, we found SARS2E-mKate2 organized into distinct ribbons in the cytoplasm (Fig. 1*A*) (44). There was some proximity in signal between SARS2E-mKate2 and the fluorescently tagged markers for the ER, ERGIC, or Golgi; however, we observed that SARS2E-mKate2 overlapped distinctly with the mitochondrial marker (Fig. 1*A*, *far right*). We observed a significant (*p* = 0.0007) increase in the average Pearson’s correlation coefficient for the MitoTracker Green marker compared with the negative control (Fig. S1, *A* and *B*) and significant increases when comparing SARS2E-mKate2 to the ER marker (*p* < 0.0001; Fig. S1, *A* and *B*) and the ER exit site marker Sec24c (*p* = 0.0156; Fig. S1, *A* and *B*). These data reveal quantitative correlations between SARS2E-mKate2, mitochondria, and certain ER membrane markers, indicating a predominantly mitochondrial and partial ER localization of E.

In further dissecting E localization, untagged E was found abundantly in mitochondria-enriched membranes as determined by cellular fractionation studies (Figs. 1*B*, S1*C*). The mean area of mitochondrial morphology was found to be smaller than that of the mock control, as measured using the Mitochondria Analyzer plugin available on ImageJ (National Institutes of Health) (Fig. 1*C*). While Pearson’s colocalization data were not entirely reflective of the visual analysis (Fig. S1, *A* and *B*), plot profiles of signal intensity against distance in the cells expressing or stained for organelle markers provided clearer insight into what was observed (Fig. 1, *D*–*G*). Plot profiles indicated little to no overlap between the signals for ER membranes and mitochondria stain (Fig. 1*D*). SARS2E-mKate2 and the ER membranes (Fig. 1*E*) display a similar phenotype of proximal but not overlapping. Figure 1*F* displays a negative control for overlaps, comparing the plasma membrane and mitochondria stains. There is a compelling overlap between the signals for SARS2E-mKate2 and mitochondria (Fig. 1*A*, *far right*, Fig. 1*G*). Altogether, these findings suggest E specificity to mitochondrial membranes and proximity to ER organelles.

### E colocalizes with Cer and impacts Cer, Cer-1-phosphate, and cholesterol levels in cell membranes

**Figure 2:**
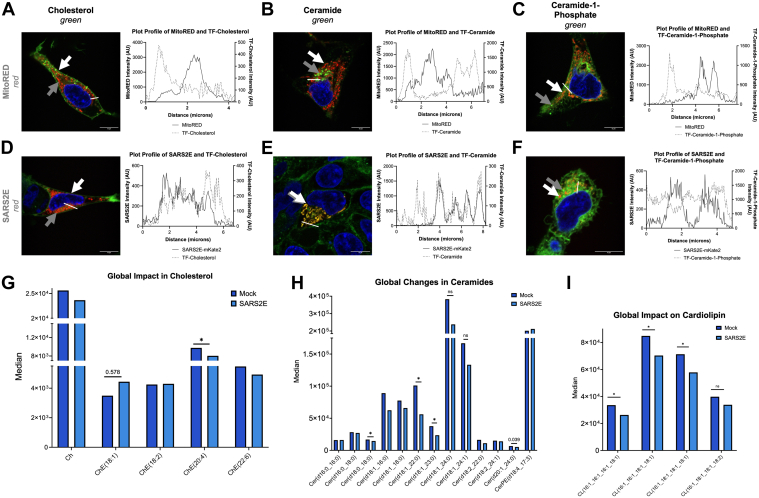
**Colocalization of SARS2E-mKate2 with ceramide and sphingolipid changes**. *A*–*F*, representative images of HEK293 cells 24 h after mock transfection (*top panel*) or post-transfection with SARS2E-mKate2 (SARS2E) (*bottom panel*). *A*, TF-cholesterol (*green channel* and *white arrow*) and MitoTracker Red FM (MitoRED) (*red channel* and *gray arrow*) (n = 18) are shown with a representative plot profile stemming from the region marked by the *white line*. *B*, TF-ceramide (*green channel* and *gray arrow*) and MitoRED (*red channel* and *gray arrow*) (n = 18) are shown with a representative plot profile from the region marked by the *white line*. *C*, TF-ceramide-1-phosphate (C1P) (*green channel* and *white arrow*) (n = 18) and MitoRED (*red channel* and *gray arrow*) with a representative plot profile from the region marked by the *white line*. *D*, SARS2E-mKate2 (*red channel* and *gray arrow*) and TF-cholesterol (*green channel* and *white arrow*) (n = 30) with a representative plot profile from the region marked by the *white line*. *E*, SARS2E-mKate2 (*red channel* and *gray arrow*) to ceramide (*green channel* and *white arrow*) (n = 55) with a representative plot profile demonstrating overlap of E and ceramide from the region marked by the *white line*. *F*, SARS2E-mKate2 (*red channel* and *gray arrow*) and TF-C1P (*green channel* and *white arrow*) (n = 30) with a representative plot profile from the region marked by the *white line*. Cells were stained with fluorescent lipids to a final concentration of 1 μM, and nuclei were counterstained with Hoechst 33342 (*blue*). Cells were stained with WGA to visualize the plasma membrane. The scale bar represents 10 μm. *G*, median fold changes in cholesterol (n = 5) with *t* test values published above the respective histogram plots ∗*p* < 0.05, ∗∗*p* < 0.01, and ∗∗∗*p* < 0.001. *H*, median fold changes in ceramide species (n = 5) with *t* test values published above the respective histogram plots ∗*p* < 0.05, ∗∗*p* < 0.01, and ∗∗∗*p* < 0.001. *I*, median fold changes in cardiolipin species (n = 5) with *t* test values published above the respective histogram plots ∗*p* < 0.05, ∗∗*p* < 0.01, and ∗∗∗*p* < 0.001. C1P, ceramide-1-phosphate; E, envelope protein; HEK293, human embryonic kidney 293 cell line; SARS2E, SARS-CoV-2 E protein (envelope protein of severe acute respiratory syndrome coronavirus 2; TF, TopFluor; WGA, wheat germ agglutinin.

Computational studies have suggested that the E protein may interact with Cer (13). To explore this in cells, HEK293 cells were transfected with the SARS2E-mKate2 construct and stained with fluorescent lipids following the methodology outlined by Shirey *et al*. (45). Mock transfection controls were carried out to compare fluorescent TopFluor lipid (TF-cholesterol, TF-Cer, or TF-ceramide-1-phosphate [TF-C1P]) localization to mitochondria (visualized with MitoTracker Red FM Dye [MitoRED]) (Fig. 2, *A*–*C*). Plot profile analyses demonstrated little overlap in the lipid and mitochondria signals (Fig. 2, *A*–*C*). In the assessment of lipid and E localization, TF-cholesterol had some overlap with E signal (Fig. 2*D*), whereas we observed a clear overlap in visual signal between SARS2E-mKate2 and TF-Cer (Fig. 2*E*). This is detailed in the representative plot profile analysis shown in Figure 2*E* and is in agreement with a model previously described for E lipid interactions (13). SARS2E-mKate2 and TF-C1P showed much less overlap, but the two signals were proximal, with the green TF-C1P signal almost enclosing the red SARS2E-mKate2 signal (Fig. 2*F*).

HEK293T cells expressing untagged E protein were analyzed *via* lipidomics (Fig. S2*A*) and compared with mock controls, all exposed to the same transfection reagents. We observed an increase in cholesterol ester (ChE) (18:1) and a decrease in ChE(20:4) (Fig. 2*G*). Figure 2*H* illustrates a widespread decline in Cer species levels upon E expression. We found significantly reduced levels of all four detectable species of cardiolipin (CL) when E was present (Fig. 2*I*). Overall, E expression significantly decreases Cer, ChE, and CL, suggesting that E perturbs host lipid metabolic pathways.

### E impacts cellular oxygen consumption

**Figure 3:**
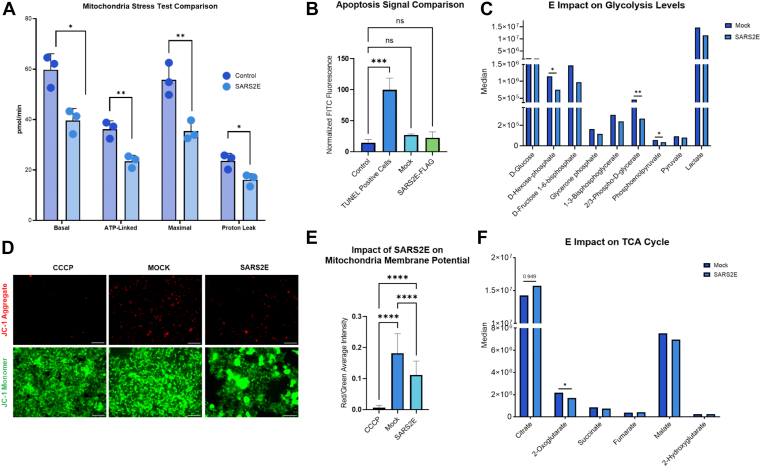
**Overall impact of E on cell death and mitochondrial dysfunction**. *A*, Seahorse XFe96 mitochondrial stress test was used to analyze OCR in HEK293 cells following SARS2E expression. Thousand cells were plated per well in an Agilent flux plate, allowed to attach, transfected with SARS2E or control plasmid using Lipofectamine 3000, and assayed following 24 h selection with Blasticidin S. OCR was measured in 18-min intervals for basal respiration and following the addition of oligomycin, FCCP, and rotenone/antimycin A. Statistics calculated *via* Student's *t* test. ∗*p* < 0.05, ∗∗*p* < 0.01. *B*, apoptosis signal was detected in transfected HEK293 cells using TUNEL assay and compared after normalization and statistically analyzed using one-way ANOVA. *C*, median fold changes in the metabolites involved in glycolysis (n = 5) with *t* test values published above the respective histogram plots. *D*, the JC-1 kit was used to analyze the impact of SARS2E expression on mitochondrial membrane potential. HEK293T cells were transfected with SARS2E untagged and, after 24 h, treated with Blasticidin S, and 48 h post-transfection, were treated with JC-1 dye and imaged CCCP (n = 18), mock (n = 12), and E (n = 23). Representative images were enhanced at 0.2%, and the median background was subtracted. The scale bar represents 50 μm. *E*, images were analyzed and quantified using ImageJ. All samples were compared by ordinary one-way ANOVA (ns, nonsignificant, *p* > 0.1234, ∗*p* < 0.0332, ∗∗*p* < 0.0021, ∗∗∗*p* < 0.0002, and ∗∗∗∗*p* < 0.0001). *F*, median fold changes in the metabolites involved in the TCA cycle (n = 5) with *t* test values published above the respective histogram plots. CCCP, carbonyl cyanide m-chlorophenylhydrazone; E, envelope protein; HEK293, human embryonic kidney 293; JC-1, 5′,6,6′-tetrachloro-1,1′,3,3′-tetraethylbenzimidazolylcarbocyanine iodide; OCR, oxygen consumption rate; SARS2E, SARS-CoV-2 E protein (envelope protein of severe acute respiratory syndrome coronavirus 2; TCA, tricarboxylic acid.

We found that the expression of SARS2E reduces HEK293T oxygen consumption rate (OCR) under all conditions of the mitochondria stress test analyzed by Seahorse (Fig. 3*A*). OCR decreases in basal respiration, proton leak, ATP linked, and maximal OCR indicate that there are major inhibitions to the electron transport chain (ETC). Maximal respiration (Fig. 3*A*) is reduced compared with controls, indicating the maximal capacity of the ETC to consume oxygen is much less with E present compared with control, when the mitochondrial membrane potential is collapsed. We did not observe significant changes in overall ATP levels when analyzing nucleotides *via* metabolomics (Fig. S2*E*).

### E impact on cell survival

When observing these changes in OCR, we performed a TUNEL assay to detect DNA fragmentation, a hallmark of apoptosis. TUNEL assay showed no significant difference in DNA fragmentation between the SARS2E-3XFLAG–transfected cells and controls at 48 h post-transfection (Fig. 3*B*). We also performed cytotoxicity and viability assays to confirm that the transfection reagents and/or treatments were not causing any sort of off-target impact on cell viability (Fig. S4). The significant impact on OCR drove our analysis of the E impact on glycolysis.

### E impact on host cell glycolysis

Expression of E led to a decrease in phosphoenolpyruvate, a central metabolite in glycolysis and gluconeogenesis that contributes to biosynthetic pathways essential for cell proliferation (Fig. 3*C*) (46). D-hexose phosphate, the phosphorylated product of glucose and a critical intermediate of the pentose phosphate pathway, was reduced (Fig. 3*C*) (47). In addition, levels of 2,3-phospho-D-glycerate, a contributor to NADPH synthesis, were significantly decreased (Fig. 3*C*). Lactate remained relatively unchanged when E is expressed (Fig. 3*C*). Together, these data indicate that E expression alters steady-state levels of key glycolytic and pentose phosphate intermediates, suggesting a broader perturbation of host central carbon metabolism.

### E depolarizes the host cell's mitochondrial membrane potential

We observed that when E is expressed, the mitochondrial membrane potential is depolarized (Fig. 3*D*). Although the effect was less pronounced than that of the positive control, carbonyl cyanide m-chlorophenylhydrazone, SARS2E expression caused a significant decrease in the red-to-green fluorescence ratio compared with mock-transfected cells (Fig. 3*E*). Overall, E causes a depolarization effect on the mitochondrial membrane potential.

### E impact on the tricarboxylic acid cycle

We observed an effect of E on the tricarboxylic acid (TCA) cycle. Specifically, an increase in glutamine levels occurred when E was expressed (Fig. S2*D*), which reflects a compensatory metabolic shift triggered by a decrease in 2-oxoglutarate, a key intermediate of the TCA cycle (Fig. 3*F*). These data demonstrate that E alone is sufficient to cause changes in glycolysis, the pentose phosphate pathway, and the TCA cycle.

### E increases cellular ROS

**Figure 4:**
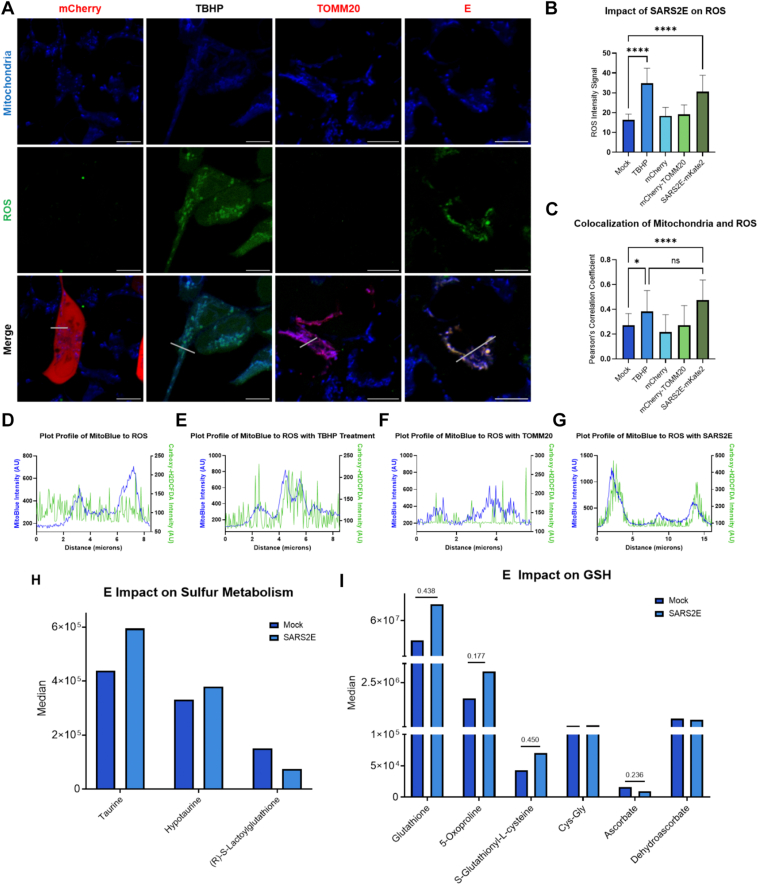
**SARS2E increases ROS production and changes ROS subcellular localization when expressed in HEK293 cells**. *A*, images of HEK293 cells transfected with mCherry vehicle, mock, TOMM20-mCherry, and SARS2E-mKate2 from *right* to *left*, respectively. Mock cells were treated with TBHP for 90 min before staining for ROS and imaging 24 h post-transfection (n = 36). The scale bar represents 10 μm. *B*, ROS intensity average was measured and compared *via* a one-way ANOVA. All samples were compared by ordinary one-way ANOVA (ns, nonsignificant, *p* > 0.1234, ∗*p* < 0.0332, ∗∗*p* < 0.0021, ∗∗∗*p* < 0.0002, and ∗∗∗∗*p* < 0.0001). *C*, Pearson’s colocalization coefficient compared with the colocalization of mitochondria (MitoBlue) and ROS signal. *D*–*G*, plot profile of signal intensity over distance defined by the *white line* on the *bottom panel* of (*A*). *H and I*, median fold changes in the number of metabolites measured *via* mass spectrometry, looking specifically at sulfur metabolism (n = 5) (*H*) and GSH homeostasis (*I*). Significant *t* test values are posted above the histogram comparison ∗*p* < 0.05, ∗∗*p* < 0.01, and ∗∗∗*p* < 0.001. HEK293, human embryonic kidney 293 cell line; ROS; reactive oxygen species; SARS2E, SARS-CoV-2 E protein (envelope protein of severe acute respiratory syndrome coronavirus 2; TBHP, *tert*-butyl hydroperoxide.

Levels of ROS were significantly increased in cells expressing SARS2E-mKate2 (Fig. 4, *A* and *B*). While the ROS signal intensity of SARS2E expression in cells was comparable to cells treated with *tert*-butyl hydroperoxide (TBHP), an inducer of ROS, the spatial distribution of ROS differed prominently (Fig. 4*C*). In SARS2E-mKate2–expressing cells, ROS staining strongly colocalized with mitochondrial markers (Fig. 4*C*), whereas TBHP-treated cells displayed a more diffuse cytosolic ROS signaling (Fig. 4*E*). Plot profile analysis further confirmed this distinction: ROS and mitochondrial signals showed strong spatial overlap in E-expressing cells (Fig. 4, *D*–*G*); the qualitative differences revealed by image analysis and profiling suggest mitochondrial retention of ROS with detection of E. These data show that E not only induces ROS increases but also leads to higher retainment of ROS in the mitochondria.

### E impact on mitochondrial metabolism

We investigated the impact that mitochondrial dysfunction may have on carnitine and fatty acid metabolism. We observed a specific reduction in AC(4-OH), a lipid involved in carnitine and fatty acid metabolism within mitochondrial membranes (Fig. S2*C*) (48). E also had a small impact on increasing taurine and hypotaurine levels while slightly decreasing S-lactoylglutathione (Fig. 4*H*), a key intermediate in the glyoxylase system. When reviewing the impact of E on diacylglycerides (DGs), we observed 36 species and found 20 of them to be reduced (Fig. S3*F*). Again, we observed a range of reductions at various chain lengths; however, the modified DG(O) species were almost entirely reduced across all chain lengths but one, DG(O12:1_22:1) (Fig. S3*F*) (49). Consistent with this, we detected subtle but notable changes in the GSH antioxidant pathway, including increases in GSH, 5-oxoproline, S-glutathionyl-L-cysteine, and cysteinylglycine (Fig. 4*I*). In contrast, levels of ascorbate (vitamin C) and its oxidized form, dehydroascorbate, were reduced (Fig. 4*I*). Although both the GSH and ascorbate pathways are functionally linked through L-cysteine metabolism, we observed no significant changes in cysteine levels (Fig. S2*D*).

### E impact on additional lipidomics

We looked further into phospholipid levels, including phosphatidylcholine (PC), lysophosphatidylcholines (LPCs), phosphatidylethanolamine (PE), and lysophosphatidylethanolamine (LPEs). PC levels were relatively unchanged, except when we start to look at chain lengths greater than 40:0, where the levels are significantly reduced when E is expressed (Fig. S3*A*). Similarly with the modified PCs, there is a range of significant reduction of levels of PC(O-36:0) through PC(O-38:3), PC(O-42:2), PC(O-42:1), and PC(O-42:2) (Fig. S3*C*). Of the 17 LPC species analyzed, 10 of them were significantly reduced when E was expressed (Fig. S2*B*).

About half of the PE species measured were found to be significantly reduced when E was expressed but not in any obvious chain length–specific pattern (Fig. S3*B*). There was a significant reduction of all three LPEs (Fig. S2*B*) when E was expressed. Both lysophosphatidylinositol 18:0 (Fig. S2*B*) and phosphatidylinositol (PI) 18:0 (Fig. S3*D*) were significantly reduced. Compared with the PC changes at longer chain lengths, there appears to be a pattern of reduction of PIs at shorter chain lengths, PI(15:0_16:1) through PI(20:3_18:0) and modified PI(O-16:0_18:1) and PI(O-16:0_20:1) (Fig. S3*D*).

For both lysophosphatidylserine (18:0) (Fig. S2*B*) and phosphatidylserine (PS) (18:0_20:4) (Fig. S3*E*), we observed significant reductions in both species when E was expressed. When observing other species of PS, we found that across the range of chain lengths, there were select species reduced, with a significant reduction in the four longest chain lengths measured, including PS(O-20:0_26:1), PS(20:0_18:1), PC(22:0_18:1), and PS(24:0_18:1) (Fig. S3*E*). Overall, these data suggest that E has a wide impact on host lipid metabolism.

## Discussion

SARS2E-mKate2 formed cytoplasmic tubular structures that resemble those previously reported for the mouse hepatitis virus, a coronavirus discovered decades before SARS-CoV-2 (50). While prior studies had established E’s association with ER-derived membranes, our data identify an additional localization site: the mitochondria. This is consistent with a prior report suggesting E serves as a bridge between the ER and mitochondria connection points (39). E appears to have an impact on the morphology of mitochondria that we hypothesize is being driven by the decrease in DG species that serve both a structural and a secondary messenger role in mitochondrial morphology and fragmentation (49, 51). While E appeared proximal to the ER, ERGIC, and Golgi membranes, colocalization was the strongest with mitochondria. No significant overlap was observed between E and SARS-CoV-2 structural proteins M or S, further supporting previous work showing E is minimally incorporated into mature virions and is not abundantly colocalizing with these proteins outside that function (10, 27). While previous work has shown that E alone elicits strong immune responses, its mitochondrial localization may play a direct role in activating mitochondrial antiviral signaling pathways (17, 52, 53). Together, these findings further support E’s complex, dual functions—supporting virion assembly and budding and independently disrupting host cell physiology and metabolism to support virion reproduction.

SARS-CoV-2 is known to impair mitochondrial function, but the specific viral factors involved have not been fully defined (40, 54). Given our observed mitochondrial localization of E, we examined its potential role in this dysfunction. E expression alone resulted in decreased levels of CL, a key lipid that maintains mitochondrial membrane structure and supports ETC organization (55). With less CL to stabilize the components of the ETC, we found evidence of ETC dysfunction in decreased OCR for all measures of cellular respiration. We hypothesize that this dysfunction contributes to the depolarization of mitochondrial membrane potential and subsequent ROS production (56, 57). While another SARS-CoV-2 viroporin, ORF3a, has been implicated in ROS production (17), our results demonstrate that E alone is sufficient to increase ROS production. There is a unique retention of ROS within mitochondria that we suspect is linked to the presence of E at mitochondrial membranes. The retention of ROS suggests intact antioxidant buffering capacity in the mitochondria (58) potentially limiting the spread of oxidative stress, which may explain the lack of apoptosis signaling despite mitochondrial depolarization.

Finding more specificity in SARS2E impact on COVID-19 infection revealed that while PS receptors facilitate viral entry (59), similar to the role of Cer in SARS-CoV-2 infection (60, 61), we observed a marked reduction in PS as well as Cer levels. In addition, the severity of COVID-19 has been correlated to the levels of PC, LPC, PE, and LPE (62), and our lipidomic analysis revealed that E expression decreases several of these lipid species. LPCs are biomarkers associated with sepsis severity and were likewise reduced in the presence of E (63). Given that LPCs and LPEs are involved in lipid droplet formation and calcium signaling, and calcium can flux through E’s viroporin channel (62), these lipid changes may further support a role for E in altering host cell lipid architecture to favor viral replication. Notably, prior cellular and lipidomic studies on SARS-CoV-2 infections detected an increase in lipid droplet formation (55) and extensive lipid metabolic changes across several variants of SARS-CoV-2 (55, 64). E lowering ChE, CL, and some lysolipids in this study is consistent with previous lipidomic studies demonstrating a reduction in ChE, CL, and lysolipids by SARS-CoV-2 infection and a reduction in CL when E was expressed in HEK293T cells (55). In contrast, the reduction in Cers by E expression is not in line with increased Cers observed for authentic SARS-CoV-2 infection in cells (55, 64). Thus, other SARS-CoV-2 proteins may be responsible for increasing Cer levels, likely *via* reduction in levels of ORMDL (a regulator of sphingolipid biosynthesis) as recently identified in SARS-CoV-2 cellular infections (64). Notably, E expression led to a reduction in multiple lipid classes and the mechanism behind such reduction is a limitation of the current study. While the number of mitochondria was not measured herein, a reduction in the number of mitochondria could be one mechanism by which multiple lipid classes were lowered by E expression. Furthermore, we speculate that E on its own may increase mitophagy, which is in part supported by the decrease in mitochondria size we detected in Figure 1*C* and the increase in mitochondrial ROS.

While E may act as a calcium channel (39, 44, 65, 66), the exact mechanism by which it disrupts mitochondrial membrane potential remains unclear. The surprising finding that ROS remains localized within mitochondria points to a complex regulation of mitochondrial redox homeostasis. E expression alone recapitulated phenotypes associated with severe COVID-19, including reduced vitamin C metabolites (67). The findings that E lowers mitochondrial OCR may be relevant to SARS-CoV-2 infection and long COVID, which has been shown to induce mitochondrial metabolic changes (64, 65, 66, 67, 68, 69, 70). Examination of mitochondrial flux analysis from long COVID syndrome (27 patients) by Seahorse analysis discovered changes in mitochondrial ATP synthase activity (69). It was hypothesized that ATP synthase activity is changed, whereby ATP is not only synthesized by but also hydrolyzed by the enzyme (69). Mitochondrial respiration has also been proposed as a biomarker of severe and long COVID (70). Peripheral blood mononuclear cells from patients were shown to have impaired mitochondrial respiration in intensive care hospitalized COVID patients and patients with long COVID (70). Furthermore, SARS-CoV-2 infections in Huh7.5 cells increased mitochondrial ROS levels, and a mitochondria-targeting scavenger compound impaired viral infection (71). Thus, the role of E in our study may be linked to the effects of E and other SARS-CoV-2 proteins on mitochondria during SARS-CoV-2 infection and contribute to long COVID symptoms.

To further analyze E's impact on mitochondrial metabolism, TCA metabolites and amino acids were quantified. The only change in amino acids was found in an elevation of glutamine levels that we hypothesize may compensate for decreased 2-oxoglutarate to sustain TCA cycle flux (39, 54). While glutamine has been proposed as a therapeutic supplement for COVID-19 (55), our data suggest that its increase here may contribute to ROS generation and mitochondrial stress. This increase may reflect enhanced glutamine catabolism to glutamate and subsequent anaplerosis (72). The decrease in TCA metabolite AC(OH) may reflect underlying disruptions in oxidative metabolism and contribute to mitochondrial ROS production (73). We hypothesize that these reductions, in combination with the reductions in levels of 2,3-phospho-D-glycerate by E alone, can contribute to hypoxic conditions (74). These modulations in mitochondrial metabolism lead us to take a closer look at glycolysis, which is frequently hijacked by viruses to boost metabolic outputs that support viral replication (75, 76).

To evaluate the impact of the SARS-CoV-2 E protein on this pathway, we measured key glycolytic intermediates. We detected increased levels of GSH, 5-oxoproline, S-glutathionyl-L-cysteine, and cysteinylglycine, alongside decreased levels of ascorbate and dehydroascorbate, all players in redox and sulfur metabolism. These changes implicate disruption of antioxidant networks, though cysteine levels remained unchanged. We observed increases in taurine and hypotaurine metabolites in the sulfur metabolism pathway that are known to modulate oxidative stress in other viral infections, such as chikungunya virus (77). Altogether, we propose that E may promote a metabolic shift toward the Warburg effect—a form of aerobic glycolysis often co-opted by viruses and cancer cells to support replication and survival (78, 79). The combination of increased glutamine, increased ROS, decreased CL, and mitochondrial membrane depolarization suggests that E contributes to a dysfunctional ETC and a metabolic environment favorable to viral propagation (80). While lactate is not changed and is a typical driver of the Warburg effect, glutamine has also been shown to serve as a carbon source for the TCA cycle anaplerosis (79, 81). Thus, E may induce changes in metabolism that support glutamine metabolism to provide replenishment to the TCA cycle.

In seeking to understand the subcellular localization of E, we uncovered a complex network of metabolic changes centered on mitochondria. Initial findings of mitochondrial dysfunction in membrane potential and ROS production led us to find underlying metabolic changes. Overall, our findings highlight the multifaceted role of the E protein in SARS-CoV-2 pathogenesis and underscore its potential as a therapeutic target in current and future coronavirus outbreaks.

## Experimental procedures

### Cultivation of cells

HEK293 cells (American Type Cell Collection) were cultivated in Dulbecco’s modified Eagle’s medium (DMEM) with 10% fetal bovine serum (FBS) and 1% penicillin–streptomycin (all reagents from ThermoFisher Scientific) at 37 °C in a 5% CO_2_ incubator. The cells were passaged two or three times per week, as required (1). Human epithelial, embryonic kidney 293T cells (American Type Culture Collection) were cultivated in the same conditions as HEK293 cells.

### Plasmids

Cells were transfected with the following SARS-CoV-2 codon-optimized plasmids: pcDNA3-SARS-CoV-2-E-mKate2 (a gift from Masayuki Yazawa, Icahn School of Medicine at Mount Sinai), pUNO1-SARS2-E (InvivoGen, catalog no.: puno1-cov2-e), pCMV 3xFlag-Envelope, and pCDNA3 GFP-SARS2M (gifts from Erica Ollmann Sapphire, La Jolla Institute of Immunology), and pcDNA3.1 Spike-GFP plasmid (GenScript Biotech; MC_0101089; a gift from Raluca Ostafe, Purdue University).

The colocalization markers and controls, pUNO1-mcs (InvivoGen, catalog no.: puno1-mcs), pGEX-KG-D4H∗-mCherry (Addgene #134604), mCherry-TOMM20-N-10 (Addgene # 55146), pmEmerald-ERGIC-53 (Addgene #170717), mEmerald-ER-3 (Addgene #54149), mEmerald-Lysosomes-20 (Addgene #54149), mEmerald-Golgi-7 (Addgene #54108), and Sec24c-eYFP (Addgene #66608).

### Transfection of cells and confocal microscopy

HEK293 cells were cultivated in DMEM + 10% FBS + 1% PS in 35-mm glass-bottom plates (Cellvis). They were transfected at 70% confluency with 2.5 μg E-mKate2 with Lipofectamine 2000 according to the manufacturer’s protocols (ThermoFisher Scientific, catalog no.: 11-668-019). After 6 h, the cells were supertransfected with 2.5 μg of plasmid pmEmerald-ERGIC-53, mEmerald-ER-3, mEmerald-20, or mEmerald-Golgi-7. Twenty-four hours post-transfection, confocal imaging was conducted using a Nikon Eclipse Ti A1Rsi instrument with NIS-elements AR software to capture 1024 × 1024 pixel resolution images at 1/8 frames/s using a 60× oil objective, detecting channels in series at the Purdue College of Pharmacy Live Cell Imaging Facility.

### Mitochondrial staining for confocal microscopy

Invitrogen MitoTracker Green (ThermoFisher, catalog no.: M7514) was resuspended to a stock concentration of 1 mM in dimethyl sulfoxide (DMSO) and was used at a final concentration of 20 nM. HEK293 cells were washed twice before staining with MitoTracker Green in Opti-MEM for 15 min at 37 °C. The nuclei were counterstained with Hoechst 33342 at 10 μM. Molecular Probes MitoTracker Red FM (ThermoFisher Scientific, catalog no.: M22425) was resuspended to a stock concentration of 1 mM in DMSO and used at a final concentration of 100 nM for 15 min at 37 °C. The stain was removed, and cells were rinsed once with PBS before imaging in fresh Opti-MEM. BioTracker 405 Blue Mitochondria Dye (MitoBlue) was resuspended to a stock concentration of 200 μM stock solution in DMSO and was used at a final concentration of 200 nM for 15 min at 37 °C. The stain was then removed, and cells were rinsed once with Opti-MEM before imaging in fresh Opti-MEM.

### Mitochondria isolation from epithelial cells

HEK293T cells were transfected with pUNO1-mcs and pUNO1-SARS2-E using Lipofectamine 3000 according to the manufacturer's instructions for 5 μg of DNA per 100 mm plate. To select for E protein, cells were incubated with DMEM + 10% FBS + 1% PS overnight before media exchange with complete DMEM treated with 10 μg/ml Blasticidin S (ThermoFisher, A1113903) for 24 h before the media were removed, cells were rinsed with room temperature Dulbecco’s PBS twice. Approximately 100 μl of cells were set aside to collect the whole cell lysate fraction for immunoblotting. Mitochondria were isolated using the Mitochondria Isolation Kit for cultured cells according to the manufacturer’s protocol (ThermoFisher Scientific, catalog no.: 89874).

### Immunoblotting

Protein samples (25 μg/well, concentration determined using the Pierce BCA Protein Assay Kit, ThermoFisher Scientific) were mixed with 6× loading dye (375 mM Tris–HCl/Tris base, 9% SDS [w/v], 50% glycerol [v/v], 9% 2-mercaptoethanol [v/v], and 0.075% bromophenol blue [w/v]) and separated by 4-20% gradient SDS-PAGE and transferred to a polyvinylidene fluoride membrane. Membranes were blocked with a 1:10 dilution of Casein Blocking Solution in Tris-buffered saline (CAS #7732-18-5) to Tris-buffered saline with Tween-20. SARS2E was visualized using SARS-CoV-2 Envelope Recombinant Rabbit Monoclonal Antibody (HL1443) (ThermoFisher, #MA5-47046), Aconitase 2 Polyclonal Antibody (ThermoFisher, PA5-19269), GM130 Polyclonal Antibody (ThermoFisher, PA1-077), GAPDH Polyclonal Antibody, and horseradish peroxidase (HRP) (ThermoFisher, PA1-987-HRP). Antibodies were visualized using appropriate secondary antibodies, Rabbit anti-Goat IgG (H+L) Secondary Antibody, HRP (ThermoFisher, 31402), and Goat anti-Rabbit IgG (H+L) Secondary Antibody, and HRP (ThermoFisher, 31460). HRP signal was visualized *via* Clarity Western ECL Substrate (Bio-Rad, 1705061).

### Fluorescent lipids live-cell confocal microscopy

TF lipids were prepared on the day of imaging as previously described (45). The lipids were resuspended in 100% ethanol and sonicated (10 s pulse, 10 s interval) 10 times. The lipids were then incubated at 37 °C for at least 30 min. Nuclei were counterstained with Hoechst 33342, and the cells were washed twice before adding lipids directly to the Opti-MEM imaging medium at a final concentration of 1 μM. Samples were incubated at 37 °C for approximately 30 min before imaging.

### High-throughput metabolomic and lipidomic sample preparation

Metabolites were extracted *via* a modified protein crash from previously described work (82, 83, 84). Extraction of metabolites from cells was as follows: Variable amounts of cold MeOH:acetonitrile (ACN):H_2_O (5:3:2, v:v:v) were added to each cell pellet so that the final extraction concentration of each sample was 2 × 10^6^ cells/ml. The samples were then vortexed at 4 °C for 30 min. Following vortexing, samples were centrifuged at 18,300*g* for 10 min at 4 °C, and the supernatant was transferred to a new autosampler vial for analysis. A portion of the extract from each sample was also combined to create a technical mixture, injected throughout the run for quality control. Lipidomics were extracted using identical preparation techniques as metabolomics, save for utilizing 50:50 MeOH:isopropyl alcohol instead of MeOH:ACN:H_2_O (5:3:2, v:v:v) as an extraction buffer.

### High-throughput metabolomics analysis

Analyses were performed as previously published *via* a modified gradient optimized for the high-throughput analysis of metabolomics (82, 83, 84). Briefly, the analytical platform employs a Q Exactive MS system (Thermo Fisher Scientific) coupled online to a Q Exactive mass spectrometer (Thermo Fisher Scientific). Metabolomics extracts were resolved over an ACQUITY UPLC BEH C18 column (2.1 × 100 mm, 1.7 μm particle size) held at 45 °C (Waters). For positive mode, mobile phase (A) 0.1% formic acid in water and mobile phase (B) 0.1% formic acid in ACN was used. For negative mode, mobile phase (A) 10 mM ammonium acetate in water and mobile phase (B) 10 mM ammonium acetate in 50:50 ACN:MeOH was used. For negative and positive mode analysis, the chromatographic gradient was as follows: 0.45 ml/min flow rate for the entire run, 0% B at 0 min, 0% B at 0.5 min, 100% B at 1.1 min, 100% B at 2.75 min, 0% B at 3 min, and 0% B at 5 min. For positive ion mode, the Exploris 120 mass spectrometer (Thermo Fisher) scanned in full mass spectrometry (MS) mode from 65 to 975*m/z* at 120,000 resolution, with 3.5 kV spray voltage, 50 sheath gas, and 10 auxiliary gas. For negative ion mode, the Exploris 120 mass spectrometer (Thermo Fisher) scanned in full MS mode from 65 to 975*m/z* at 120,000 resolution, with 3.4 kV spray voltage, 50 sheath gas, and 10 auxiliary gas. Calibration was performed prior to analysis using the Pierce Positive and Negative Ion Calibration Solutions (Thermo Fisher Scientific).

### High-throughput lipidomic analysis

Lipid extracts were analyzed (5 μl per injection) on a Thermo Q Exactive MS/Q Exactive MS system using a modified previously described 5 min lipidomics gradient and a Kinetex C18 column (30 × 2.1 mm, 1.7 μm, Phenomenex) held at 50 °C. Mobile phase (A): 25:75 ACN:H_2_O with 5 mM ammonium acetate; mobile phase (B): 90:10 isopropyl alcohol:ACN with 5 mM ammonium acetate. The gradient and flow rate were as follows for negative mode: 0.3 ml/min of 10% B at 0 min, 0.3 ml/min of 95% B at 3 min, 0.3 ml/min of 95% B at 4.2 min, 0.45 ml/min 10% B at 4.3 min, 0.4 ml/min of 10% B at 4.9 min, and 0.3 ml/min of 10% B at 5 min. The gradient and flow rate were as follows for positive mode: 0.3 ml/min of 30% B at 0 min, 0.3 ml/min of 100% B at 3 min, 0.3 ml/min of 100% B at 4.2 min, 0.45 ml/min 30% B at 4.3 min, 0.4 ml/min of 30% B at 4.9 min, and 0.3 ml/min of 30% B at 5 min. Samples were run in positive and negative ion modes (both electrospray ionization, separate runs) at 125 to 1500 *m/z* and 70,000 resolution, 4 kV spray voltage, 45 sheath gas, and 25 auxiliary gas. The MS was run in data-dependent acquisition mode (ddMS2) with top10 fragmentation. Raw MS data files were searched using LipidSearch, version 5.0 (Thermo Fisher Scientific).

### High-throughput metabolomic and lipidomic data analysis

Acquired data were converted from raw to the mzXML file format using RawConverter. Analysis was done using El MAVEN, an open-source software program for linoleic acid quantification. All other lipid assignments and peak integrations were performed using LipidSearch, version 5.0. Samples were analyzed in randomized order with a technical mixture injected interspersed throughout the run to qualify instrument performance.

### Seahorse mitochondria stress test

An Agilent XFe96 Seahorse mitochondrial stress test was conducted to measure both OCR and extracellular acidification rate under various conditions. HEK293 cells were seeded at 1000 cells per well in an XFe96 microwell plate. Following attachment, cells were transfected with the indicated plasmids using Lipofectamine 3000 according to the manufacturer’s suggested parameters. Media were removed 24 h later and replaced with selection media containing 10 ng/μl Blasticidin S. After 24 h, cells were switched into Seahorse media (Agilent RPMI supplemented with 2 mM glutamine, 1 mM sodium pyruvate, and 10 mM glucose) and allowed to equilibrate in a CO_2_-free incubator for 30 min. During equilibration, a prehydrated (24 h with Agilent Calibrant) XFe96 sensor cartridge was loaded with oligomycin (1 μM final), FCCP (1 μM final), and rotenone/antimycin A (0.5 μM final). OCR and extracellular acidification rate measurements were taken in 18-min intervals for basal respiration and following the addition of oligomycin, FCCP, and aotenone/antimycin A.

### Apoptosis TUNEL assay

HEK293 cells were transfected using a 1:3 ratio of plasmid DNA to polyethyleneimine (PEI), which was thawed at room temperature before combining 50 μl Opti-MEM with 16 μg DNA per 100 mm plate. Cells were incubated at 37 °C in 5% CO_2_ for 4 to 6 h before media exchange. Forty-eight hours post-transfection of SARS2E-3XFLAG, 1 to 5 × 10^6^ cells were collected in 1X PBS and washed by centrifugation at 300*g* for 5 min before resuspending them in 0.5 ml PBS and repeating the centrifugation and pelleting steps. The cells were then resuspended in 0.5 ml 70% ethanol and incubated for 30 min on ice with careful resuspension every 10 min. A TUNEL assay was then conducted using the Abcam TUNEL kit according to the manufacturer’s instructions (Abcam). The cells were then analyzed by flow cytometry using a BD Fortessa Cell Analyzer at excitation and emission wavelengths of 488 and 520 nm for FITC, with data presented as fluorescence values per 10,000 events. Statistical significance was determined by one-way ANOVA.

### Mitochondrial membrane potential visualization

JC-1 (5′,6,6′-tetrachloro-1,1′,3,3′-tetraethylbenzimidazolylcarbocyanine iodide) Dye (Mitochondrial Membrane Potential Probe) (ThermoFisher Scientific, #T3168) stock solution was prepared at 2.5 mg/ml in DMSO, divided into single-use aliquots, and stored at −20 °C. JC-1 was added to Opti-MEM to a final concentration of 2.5 μg/ml after washing cells gently twice with Opti-MEM. Cells were incubated with JC-1 for 30 min at 37 °C in 5% CO_2_. Cells were washed once, and Hoechst 33342 was added to a final dilution of 1:200,000 in Opti-MEM before imaging on KEYENCE BZ-X800 with Plan Fluor 40× objective. JC-1 monomer excites at 485 nm and emits at 530 nm, whereas JC-1 aggregate excites at 535 nm and emits at 590 nm. The ratio of red to green was determined using ImageJ to determine quantitatively the relative amount of depolarization happening at the mitochondrial membranes.

### Visualization of ROS

Cells were incubated with 100 μM TBHP for 90 min at 37 °C. The cells were washed once with PBS before proceeding to ROS and mitochondria staining. A 10 mM stock solution of carboxy-H2DCFDA (#C400, ThermoFisher Scientific) in DMSO was diluted to a final concentration of 10 μM in Opti-MEM. Cells were washed once with warm PBS before the carboxy-H2DCFDA solution was added. The cells were incubated for 30 min at 37 °C and washed once with warm PBS before imaging in fresh Opti-MEM. ROS intensities were measured using ImageJ.

### Immunofluorescence assay

HEK293 cells were transfected at 70% confluency in 24-well plates with 0.5 μg of SARS2E-3XFLAG using Lipofectamine 2000 according to the manufacturer’s instructions. Twenty-four hours post-transfection, cells were fixed with 4% paraformaldehyde and washed with 1X PBS. Cells were permeabilized with 0.2% Triton X-100 and blocked with 3% bovine serum albumin. Cells were incubated with antibodies for 1 h, followed by three washes with 1X PBS, starting with DYKDDDK Tag Antibody (1:5000 dilution, Invitrogen, 14-6681-82), followed by Goat anti-Mouse IgG H&L (Alexa Fluor 647) antibody (1:10,000 dilution, Abcam, ab150115). For organelle immunodetection, anti-GM130 (Abcam, ab52649), anti-LAMP1 (Abcam, ab278043), anti-Calnexin (Abcam, ab22595), and anti-HSP60 (ThermoFisher Scientific, catalog no.: PA5-34760) antibodies were used at a 1:100 dilution, followed by Donkey anti-Rabbit IgG H&L (Alexa Fluor 488) (1:1000 dilution, Abcam, ab150073). Nuclei were stained with Hoechst 33342 at 0.1 μm and imaged on a Nikon Ti A1si confocal microscope with a 60x oil objective.

### Colocalization and statistical analysis

Image processing in ImageJ was conducted using Just Another Colocalization Plugin (Fabrice Cordelières) after removing the background by taking the mean of the signal and subtracting that value. The contrast was enhanced to 0.1. Pearson’s and Manders’ coefficients were calculated using the Just Another Colocalization Plugin (85). Image analysis was validated using GraphPad Prism (GraphPad Prism Software), version 9 and one-way ANOVA to determine the significance of colocalization when comparing signals in different channels. Outliers were removed using GraphPad Prism, version 9, to 5%. The statistical relevance of the proximity between the green and red signals in each image was determined using Pearson’s coefficient. This provided a correlation value for the degree of overlap, ranging from −1, meaning complete separation, to +1, meaning perfect pixel-level correlation (85).

### Cytotoxicity assay and cell viability assay

Cell viability under different transfection and selection conditions used in this study was measured using the CyQUANT MTT Cell Proliferation Assay Kit (ThermoFisher Scientific, V13154) following the manufacturer’s instructions. Cytotoxicity under different transfection and selection conditions was measured using the CyQUANT Cytotoxicity Assay kit (ThermoFisher Scientific, V23111) following the manufacturer’s instructions. Absorbance (540 nm) or fluorescence (excitation 540 nm, emission 560–580 nm) was measured using a microplate reader, respectively. Values were corrected using a fully lysed control and normalized to untreated cells to calculate relative cell viability. HEK293 or HEK293T cells were seeded in 96-well plates. HEK293 cells were treated 24 h before analysis with a mock transfection with Lipofectamine 2000, transfection of SARS2E-3XFLAG with Lipofectamine 2000, or transfection of E-mKate2 with Lipofectamine 2000 or treated 48 h before analysis with a mock transfection with PEI or transfection of SARS2E-3XFLAG with PEI. HEK293T cells were treated 48 h before analysis with a mock transfection with Lipofectamine 2000, transfection of pUNO1-mcs (InvivoGen) using Lipofectamine 2000, or transfection of pUNO1-SARS2-E using Lipofectamine 2000 and treated 24 h before analysis with 10 μg/ml Blasticidin S.

## Data availability

All data are in the article and supporting information.

## Supporting information

This article contains supporting information.

## Conflict of interest

The authors declare that they have no conflicts of interest with the contents of this article.

## References

[1] Plescia C.B., David E.A., Patra D., Sengupta R., Amiar S., Su Y. (2021). SARS-CoV-2 viral budding and entry can be modeled using BSL-2 level virus-like particles. J. Biol. Chem..

[2] Jackson C.B., Farzan M., Chen B., Choe H. (2022). Mechanisms of SARS-CoV-2 entry into cells. Nat. Rev. Mol. Cell Biol..

[3] Bianchi M., Benvenuto D., Giovanetti M., Angeletti S., Ciccozzi M., Pascarella S. (2020). Sars-CoV-2 envelope and membrane proteins: structural differences linked to virus characteristics?. Biomed. Res. Int..

[4] V’kovski P., Kratzel A., Steiner S., Stalder H., Thiel V. (2021). Coronavirus biology and replication: implications for SARS-CoV-2. Nat. Rev. Microbiol..

[5] Han Y., Zhou H., Liu C., Wang W., Qin Y., Chen M. (2024). SARS-CoV-2 N protein coordinates viral particle assembly through multiple domains. J. Virol..

[6] Miserey-Lenkei S., Trajkovic K., D’Ambrosio J.M., Patel A.J., Čopič A., Mathur P. (2021). A comprehensive library of fluorescent constructs of SARS-CoV-2 proteins and their initial characterisation in different cell types. Biol. Cell..

[7] Schoeman D., Fielding B.C. (2019). Coronavirus envelope protein: current knowledge. Virol. J..

[8] Wang W.-A., Carreras-Sureda A., Demaurex N. (2023). SARS-CoV-2 infection alkalinizes the ERGIC and lysosomes through the viroporin activity of the viral envelope protein. J. Cell Sci..

[9] Zhou S., Lv P., Li M., Chen Z., Xin H., Reilly S. (2023). SARS-CoV-2 E protein: pathogenesis and potential therapeutic development. Biomed. Pharmacother..

[10] Boson B., Legros V., Zhou B., Siret E., Mathieu C., Cosset F.-L. (2020). The SARS-CoV-2 envelope and membrane proteins modulate maturation and retention of the spike protein, allowing assembly of virus-like particles. J. Biol. Chem..

[11] Nieto-Torres J.L., Dediego M.L., Alvarez E., Jiménez-Guardeño J.M., Regla-Nava J.A., Llorente M. (2011). Subcellular location and topology of severe acute respiratory syndrome coronavirus envelope protein. Virology.

[12] Ghosh S., Dellibovi-Ragheb T.A., Kerviel A., Pak E., Qiu Q., Fisher M. (2020). β-Coronaviruses use lysosomes for egress instead of the biosynthetic secretory pathway. Cell.

[13] Sarkar M., Saha S. (2020). Structural insight into the role of novel SARS-CoV-2 E protein: a potential target for vaccine development and other therapeutic strategies. PLoS One.

[14] Kuzmin A., Orekhov P., Astashkin R., Gordeliy V., Gushchin I. (2022). Structure and dynamics of the SARS-CoV-2 envelope protein monomer. Proteins Struct. Funct. Bioinforma..

[15] Cao Y., Yang R., Lee I., Zhang W., Sun J., Wang W. (2021). Characterization of the SARS-CoV-2 E protein: Sequence, structure, viroporin, and inhibitors. Protein Sci..

[16] Castaño-Rodriguez C., Honrubia J., Gutiérrez-Álvarez J., DeDiego M., Nieto-Torres J.L., Jimenez-Guardeño J.M. (2018). Role of severe acute respiratory syndrome coronavirus viroporins E, 3a, and 8a in replication and pathogenesis. mBio.

[17] Guarnieri J.W., Angelin A., Murdock D.G., Schaefer P., Portluri P., Lie T. (2023). SARS-COV-2 viroporins activate the NLRP3-inflammasome by the mitochondrial permeability transition pore. Front. Immunol..

[18] Geanes E.S., McLennan R., Pierce S.H., Menden H.L., Paul O., Sampath V. (2024). SARS-CoV-2 envelope protein regulates innate immune tolerance. iScience.

[19] Zheng M., Karki R., Williams E.P., Yang D., Fitzpatrick E., Vogel P. (2021). TLR2 senses the SARS-CoV-2 envelope protein to produce inflammatory cytokines. Nat. Immunol..

[20] Xia B., Shen X., He Y., Pan X., Liu F.-L., Wang Y. (2021). SARS-CoV-2 envelope protein causes acute respiratory distress syndrome (ARDS)-like pathological damages and constitutes an antiviral target. Cell Res..

[21] Schoeman D., Fielding B.C. (2020). Is there a link between the pathogenic human coronavirus envelope protein and immunopathology? A review of the literature. Front. Microbiol..

[22] Fehr A.R., Perlman S. (2015). Coronaviruses: an overview of their replication and pathogenesis. Coronaviruses.

[23] Gorkhali R., Koirala P., Rijal S., Mainali A., Baral A., Bhattarai H.K. (2021). Structure and function of major SARS-CoV-2 and SARS-CoV proteins. Bioinforma. Biol. Insights..

[24] Chen S.-C., Lo S.-Y., Ma H.-C., Li H.-C. (2009). Expression and membrane integration of SARS-CoV E protein and its interaction with M protein. Virus Genes.

[25] Ruch T.R., Machamer C.E. (2012). The coronavirus E protein: assembly and beyond. Viruses.

[26] Schoeman D., Cloete R., Fielding B.C. (2022). The flexible, extended coil of the PDZ-binding motif of the three deadly human coronavirus E proteins plays a role in pathogenicity. Viruses.

[27] Santos-Mendoza T. (2023). The envelope (E) protein of SARS-CoV-2 as a pharmacological target. Viruses.

[28] Chai J., Cai Y., Pang C., Wang L., McSweeney S., Shanklin J. (2021). Structural basis for SARS-CoV-2 envelope protein recognition of human cell junction protein PALS1. Nat. Commun..

[29] Toto A., Ma S., Malagrinò F., Visconti L., Pagano L., Stromgaard K. (2020). Comparing the binding properties of peptides mimicking the envelope protein of SARS-CoV and SARS-CoV-2 to the PDZ domain of the tight junction-associated PALS1 protein. Protein Sci..

[30] Jimenez-Guardeño J.M., Nieto-Torres J.L., DeDiego M.L., Regla-Nava J.A., Fernandez-Delgado R., Castaño-Rodriguez C. (2014). The PDZ-binding motif of severe acute respiratory syndrome coronavirus envelope protein is a determinant of viral pathogenesis. PLoS Pathog..

[31] Gibson P.G., Qin L., Puah S.H. (2020). COVID-19 acute respiratory distress syndrome (ARDS): clinical features and differences from typical pre-COVID-19 ARDS. Med. J. Aust..

[32] Ghosh A., Pithadia A.S., Bhat J., Bera S., Midya A., Fierke C.A. (2015). Self-assembly of a nine-residue amyloid-forming peptide fragment of SARS corona virus E-protein: mechanism of self aggregation and amyloid-inhibition of hIAPP. Biochemistry.

[33] Ghosh A., Bhattacharyya D., Bhunia A. (2018). Structural insights of a self-assembling 9-residue peptide from the C-terminal tail of the SARS corona virus E-protein in DPC and SDS micelles: a combined high and low resolution spectroscopic study. Biochim. Biophys. Acta Biomembr..

[34] Mukherjee S., Bhattacharyya D., Bhunia A. (2020). Host-membrane interacting interface of the SARS coronavirus envelope protein: immense functional potential of C-terminal domain. Biophys. Chem..

[35] Miura K., Suzuki Y., Ishida K., Arakawa M., Wu H., Fujioka Y. (2023). Distinct motifs in the E protein are required for SARS-CoV-2 virus particle formation and lysosomal deacidification in host cells. J. Virol..

[36] Mandala V.S., McKay M.J., Shcherbakov A.A., Dregni A.J., Kolocouris A., Hong M. (2020). Structure and drug binding of the SARS-CoV-2 envelope protein in phospholipid bilayers. Res. Sq.

[37] Verdiá-Báguena C., Aguilella V.M., Queralt-Martín M., Alcaraz A. (2021). Transport mechanisms of SARS-CoV-E viroporin in calcium solutions: lipid-dependent anomalous mole fraction effect and regulation of pore conductance. Biochim. Biophys. Acta Biomembr..

[38] Pearson G.J., Mears H.V., Broncel M., Snijders A.P., Bauer D.L.V., Carlton J.G. (2024). ER-export and ARFRP1/AP-1–dependent delivery of SARS-CoV-2 envelope to lysosomes controls late stages of viral replication. Sci. Adv..

[39] Poggio E., Vallese F., Hartel A.J.W., Morgenstern T.J., Kanner S.A., Rauh O. (2023). Perturbation of the host cell Ca2+ homeostasis and ER-mitochondria contact sites by the SARS-CoV-2 structural proteins E and M. Cell Death Dis..

[40] Shang C., Liu Z., Zhu Y., Lu J., Ge C., Zhang C. (2022). SARS-CoV-2 causes mitochondrial dysfunction and mitophagy impairment. Front. Microbiol..

[41] Khodadoust M.M. (2021). Inferring a causal relationship between ceramide levels and COVID-19 respiratory distress | scientific reports. Sci. Rep..

[42] Alonso A., Goñi F.M. (2018). The physical properties of ceramides in membranes. Annu. Rev. Biophys..

[43] Lee I.-H., Kai H., Carlson L.-A., Groves J.T., Hurley J.H. (2015). Negative membrane curvature catalyzes nucleation of endosomal sorting complex required for transport (ESCRT)-III assembly. Proc. Natl. Acad. Sci. U. S. A..

[44] Cabrera-Garcia D., Bekdash R., Abbott G.W., Yazawa M., Harrison N.L. (2021). The envelope protein of SARS-CoV-2 increases intra-Golgi pH and forms a cation channel that is regulated by pH. J. Physiol..

[45] Shirey C.M., Ward K.E., Stahelin R.V. (2016). Investigation of the biophysical properties of a fluorescently modified ceramide-1-phosphate. Chem. Phys. Lipids.

[46] Yang J., Kalhan S.C., Hanson R.W. (2009). What is the metabolic role of phosphoenolpyruvate carboxykinase?. J. Biol. Chem..

[47] Aziz H., Mohiuddin S.S. (2025). StatPearls.

[48] Qu Q., Zeng F., Liu X., Wang Q.J., Deng F. (2016). Fatty acid oxidation and carnitine palmitoyltransferase I: emerging therapeutic targets in cancer. Cell Death Dis..

[49] Eichmann T.O., Lass A. (2015). DAG tales: the multiple faces of diacylglycerol—stereochemistry, metabolism, and signaling. Cell. Mol. Life Sci..

[50] Raamsman M.J., Locker J.K., de Hooge A., de Vries A.A., Griffiths G., Vennema H. (2000). Characterization of the coronavirus mouse hepatitis virus strain A59 small membrane protein E. J. Virol..

[51] Pemberton J.G., Roy K., Kim Y.J., Fischer T.D., Joshi V., Ferrer E. (2025). Acute diacylglycerol production activates critical membrane-shaping proteins leading to mitochondrial tubulation and fission. Nat. Commun..

[52] Fu Y.-Z., Wang S.-Y., Zheng Z.-Q., Yi H., Li W.-W., Xu Z.-S. (2021). SARS-CoV-2 membrane glycoprotein M antagonizes the MAVS-mediated innate antiviral response. Cell. Mol. Immunol..

[53] Wu M., Pei Z., Long G., Chen H., Jia Z., Xia W. (2023). Mitochondrial antiviral signaling protein: a potential therapeutic target in renal disease. Front. Immunol..

[54] Saleh J., Peyssonnaux C., Singh K.K., Edeas M. (2020). Mitochondria and microbiota dysfunction in COVID-19 pathogenesis. Mitochondrion.

[55] Farley S.E., Kyle J.E., Leier H.C., Bramer L.M., Weinstein J.B., Bates T.A. (2022). A global lipid map reveals host dependency factors conserved across SARS-CoV-2 variants. Nat. Commun..

[56] Paradies G., Paradies V., Ruggiero F.M., Petrosillo G. (2019). Role of cardiolipin in mitochondrial function and dynamics in health and disease: molecular and pharmacological aspects. Cells.

[57] Choi S.-Y., Gonzalvez F., Jenkins G.M., Slomianny C., Chretien D., Arnoult D. (2007). Cardiolipin deficiency releases cytochrome c from the inner mitochondrial membrane and accelerates stimuli-elicited apoptosis. Cell Death Differ..

[58] Dan Dunn J., Alvarez L.A., Zhang X., Soldati T. (2015). Reactive oxygen species and mitochondria: a nexus of cellular homeostasis. Redox Biol..

[59] Bohan D., Ert H.V., Ruggio N., Rogers K.J., Badreddine M., Briseño J.A.A. (2021). Phosphatidylserine receptors enhance SARS-CoV-2 infection. PLoS Pathog..

[60] Beckmann N., Becker K.A. (2021). Ceramide and related molecules in viral infections. Int. J. Mol. Sci..

[61] Kornhuber J., Hoertel N., Gulbins E. (2022). The acid sphingomyelinase/ceramide system in COVID-19. Mol. Psychiatry.

[62] Wei J., Liu X., Xiao W., Lu J., Guan L., Fang Z. (2023). Phospholipid remodeling and its derivatives are associated with COVID-19 severity. J. Allergy Clin. Immunol..

[63] Takatera A., Takeuchi A., Saiki K., Morioka I., Yokoyama N., Matsuo M. (2007). Blood lysophosphatidylcholine (LPC) levels and characteristic molecular species in neonates: prolonged low blood LPC levels in very low birth weight infants. Pediatr. Res..

[64] Farley S.E., Kyle J.E., Jahn H., Bramer L.M., Piehowski P.D., Shepmoes A.A. (2025). Integrated lipidomic and proteomic profiling reveals metabolic network disruption by SARS-CoV-2 variants. J. Lipid Res..

[65] Bhowal C., Ghosh S., Ghatak D., De R. (2023). Pathophysiological involvement of host mitochondria in SARS-CoV-2 infection that causes COVID-19: a comprehensive evidential insight. Mol. Cell. Biochem..

[66] Medeiros-Silva J., Somberg N.H., Wang H.K., McKay M.J., Mandala V.S., Dregni A.J. (2022). pH- and calcium-dependent aromatic network in the SARS-CoV-2 envelope protein. J. Am. Chem. Soc..

[67] Patterson T., Isales C.M., Fulzele S. (2021). Low level of vitamin C and dysregulation of vitamin C transporter might be involved in the severity of COVID-19 infection. Aging Dis..

[68] Yeung-Luk B.H., Narayanan G.A., Ghosh B., Wally A., Lee E., Mokaya M. (2023). SARS-CoV-2 infection alters mitochondrial and cytoskeletal function in human respiratory epithelial cells mediated by expression of spike protein. mBio.

[69] Macnaughtan J., Chau K.-Y., Brennan E., Toffoli M., Spinazzola A., Hillman T., Heightman M., Schapira A.H.V. (2025). Mitochondrial function is impaired in long COVID patients. Ann. Med..

[70] Charles A.-L., Debrut L., Oulehri W., Vincent V., Delagreverie H., Asael P. (2025). Impaired peripheral blood mononuclear cell (PBMC) mitochondrial respiration is associated with mortality and long COVID syndrome severity in COVID-19 patients. Int. J. Mol. Sci..

[71] Cevallos C., Jarmoluk P., Sviercz F., Freiberger R.N., López C.A.M., Delpino M.V. (2025). Ferroptosis and mitochondrial ROS are central to SARS-CoV-2-induced hepatocyte death. Front. Cell. Infect. Microbiol..

[72] Fletcher S.C., Coleman M.L. (2020). Human 2-oxoglutarate-dependent oxygenases: nutrient sensors, stress responders, and disease mediators. Biochem. Soc. Trans..

[73] Beutler E., Levine R., Luft R. (1972).

[74] Rogers S.C., Said A., Corcuera D., McLaughlin D., Kell P., Doctor A. (2009). Hypoxia limits antioxidant capacity in red blood cells by altering glycolytic pathway dominance. FASEB J..

[75] Li J., Wang Y., Deng H., Li S., Qiu H.-J. (2023). Cellular metabolism hijacked by viruses for immunoevasion: potential antiviral targets. Front. Immunol..

[76] Chen P., Wu M., He Y., Jiang B., He M.-L. (2023). Metabolic alterations upon SARS-CoV-2 infection and potential therapeutic targets against coronavirus infection. Signal Transduct. Target. Ther..

[77] Kumar A., Shrinet J., Sunil S. (2023). Chikungunya virus infection in Aedes aegypti is modulated by L-cysteine, taurine, hypotaurine and glutathione metabolism. Plos Negl. Trop. Dis..

[78] Chen P., Wu M., He Y., Jiang B., He M.-L. (2023). Metabolic alterations upon SARS-CoV-2 infection and potential therapeutic targets against coronavirus infection. Signal Transduct. Target. Ther..

[79] Icard P., Lincet H., Wu Z., Coquerel A., Forgez P., Alifano M. (2021). The key role of Warburg effect in SARS-CoV-2 replication and associated inflammatory response. Biochimie.

[80] Begum H.M., Shen K. (2023). Intracellular and microenvironmental regulation of mitochondrial membrane potential in cancer cells. Wires Mech. Dis..

[81] Smith B., Schafer X.L., Ambeskovic A., Spencer C.M., Land H., Munger J. (2016). Addiction to coupling of the warburg effect with glutamine catabolism in cancer cells. Cell Rep.

[82] Reisz J.A., Zheng C., D’Alessandro A., Nemkov T., D’Alessandro A. (2019). High-Throughput Metabolomics: Methods and Protocols.

[83] Nemkov T., Reisz J.A., Gehrke S., Hansen K.C., D’Alessandro A., D’Alessandro A. (2019). High-Throughput Metabolomics: Methods and Protocols.

[84] Nemkov T., Hansen K.C., D’Alessandro A. (2017). A three-minute method for high-throughput quantitative metabolomics and quantitative tracing experiments of central carbon and nitrogen pathways. Rapid Commun. Mass Spectrom..

[85] Manders E.M.M., Verbeek F.J., Aten J.A. (1993). Measurement of co-localization of objects in dual-colour confocal images. J. Microsc..

