# Supplementary material for: SARS-CoV-2 envelope protein mitochondrial localization reveals host metabolic disruption
Source: J Biol Chem. 2026 Apr 1;302(5):111419. doi: 10.1016/j.jbc.2026.111419 (PMC13133935; doi:10.1016/j.jbc.2026.111419)
Supplement: Supplementary Material [file mmc1.docx]

**Supporting Information**

SARS-CoV-2 envelope protein mitochondrial localization reveals host metabolic disruption

Emily A. David^1,2^, Elijah J. Bass^1^, Hannah M. Woods^1^, Daniel Stephenson^2^, Kellyann Román-Cruz^1^, Charles E. Chalfant^2,3^, Robert V. Stahelin^1,*^

^1^Borch Department of Medicinal Chemistry and Molecular Pharmacology, Purdue College of Pharmacy, Purdue Institute of Inflammation, Immunology, and Infectious Disease, Purdue University, West Lafayette, IN, USA.

^2^*Department of Medicine, University of Virginia-School of Medicine, Charlottesville, VA 22903, USA.*

*^3^Research Service, Richmond Veterans Administration Medical Center, Richmond, VA 23298, USA*

*To whom correspondence should be addressed: Robert V. Stahelin, Borch Department of Medicinal Chemistry and Molecular Pharmacology, Purdue University, 207 S. Martin Jischke Drive, West Lafayette, IN 47907, USA. TEL: 1-765-494-4152; Email: [rstaheli@purdue.edu](mailto:rstaheli@purdue.edu)

Short title: SARS-CoV-2 E protein impacts the mitochondria

Keywords: cellular localization, envelope protein, metabolism, membrane potential, mitochondria, reactive oxygen species, SARS-CoV-2

**
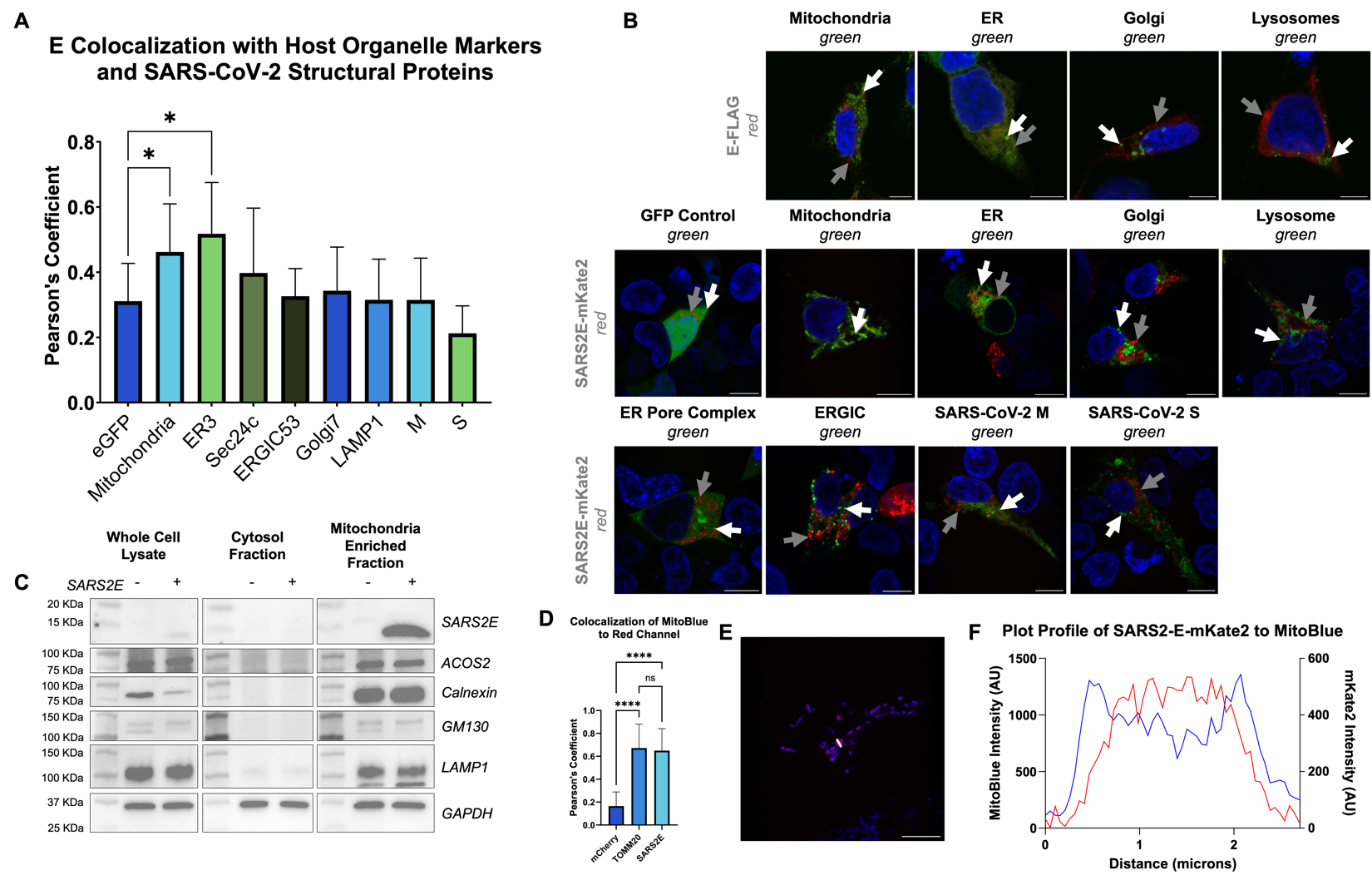
**

**Supplemental Figure 1: Broad Colocalization Analysis of SARS2E with cellular organelle markers, SARS-CoV-2 structural proteins, and mitochondrial stains.** HEK293 cells transfected with SARS2E-mKate2 and cellular markers, including EGFP (n=26), MitoTracker™ Green (n=19), ER-mEmerald3 (n=26), ER-pore complex Sec24c-GFP (n=19), ERGIC53-mEmerald3 (n=29), Golgi7-mEmerald (n=35), mEmerald-LAMP1 (n=19), GFP-SARS2M (n=18), and GFP-SARS2S (n=19) were analyzed by confocal microscopy 24 h post-transfection and counterstained with Hoechst 33342. *A*, Colocalization was measured using Pearson’s Coefficient for cell imaging represented in panel B. *B*, The top panel is representative fixed cell images of HEK293 cells transfected with E-Flag (and detected by IFA via antibody staining red or gray arrows) and organelle markers for ER (white arrows or green color), Golgi (white arrows or green color), or lysosomes (white arrows or green color). The middle and bottom panels are representative live cell images of HEK293 cells transfected with SARS-CoV-2 E-mKate2 (gray arrows or red color) and organelle markers or SARS-CoV-2 structural proteins including mitochondria (white arrows or green color), ER (white arrows or green color), Golgi (white arrows or green color), lysosomes (white arrows or green color), ER pore complex (white arrows or green color), ERGIC (white arrows or green color), SARS-CoV-2 M (white arrows or green color), or SARS-CoV-2 S (white arrows or green color) are shown. Also note, for comparison to Figure 1A we have included the same GFP control image in this panel. Scale bar = 10 µm. *C*, Mitochondria isolation from HEK293T cells transfected with untagged SARS2E and mock plasmid after 24 h Blasticidin selection (n of 1 represented here, n of 2 is in Figure 1, n=3 in total) and blotted for SARS2E, ACOS2 (mitochondria), calnexin (ER), GM130 (Golgi), LAMP1 (lysosomes), and GAPDH (loading control). A molecular weight marker ladder (kDa marked on left side of panel) is run with each E and mock plasmid condition. *D*, Pearson’s coefficient was used to measure the colocalization specificity of the images represented in *E*, mitochondria stain, MitoBlue, 24 h after HEK293 cells were transfected with mCherry, OMM protein TOMM20-mCherry, and SARS2E-mKate2. All samples were compared by ordinary one-way analysis of variance (ANOVA; ns = nonsignificant, p>0.1234, *p<0.0332, **p<0.0021, ***p<0.0002, ****p<0.0001). *E*, HEK293 cells were transfected with SARS2E-mKate2 and 24 h post-transfection, stained with MitoBlue. *E*, Plot profile on intensity from representative line in E. Scale bar = 10 µm.

**
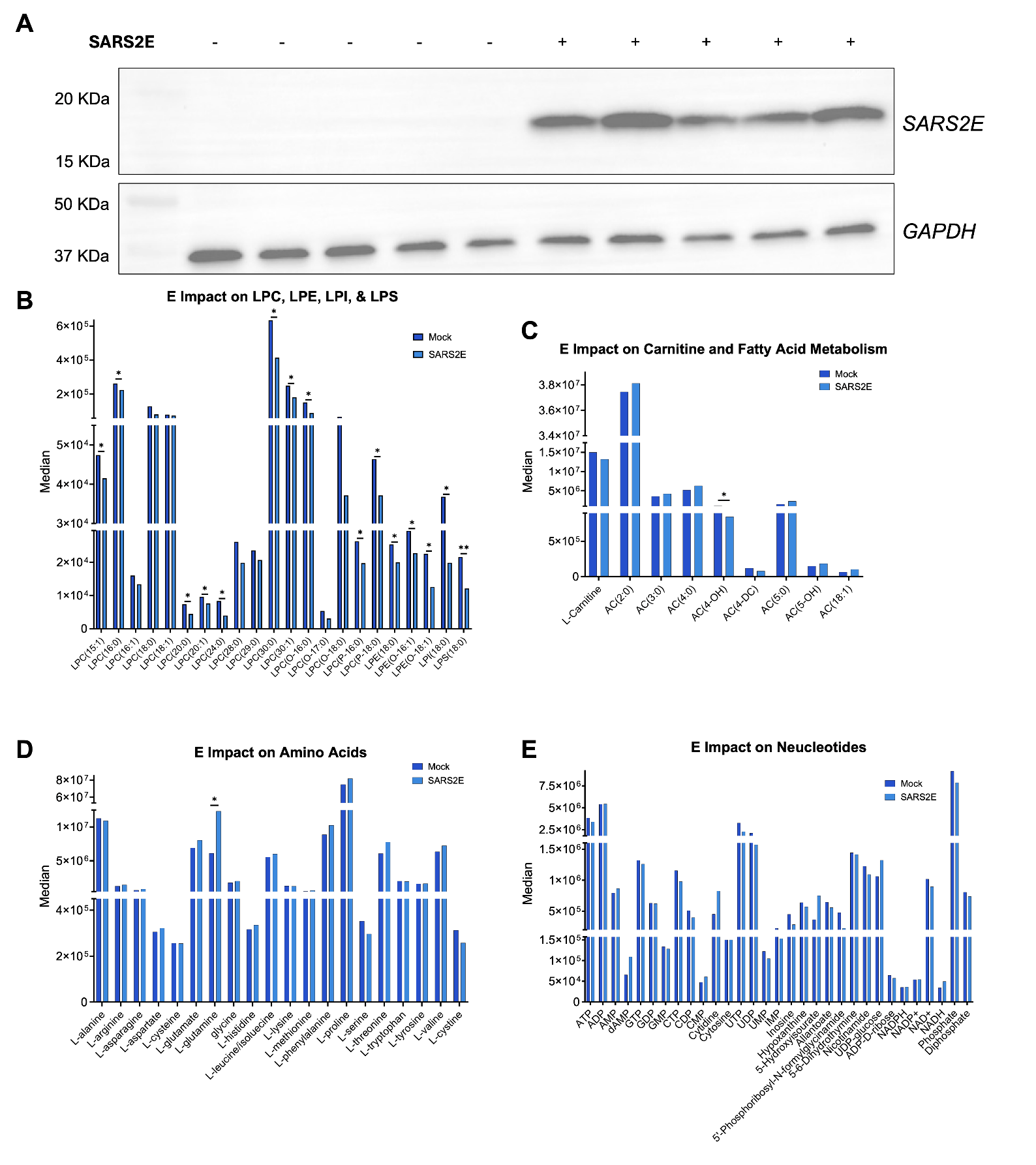
Supplemental Figure 2. Confirmation of E expression and impact of SARS2E on the LPCs, carnitine and fatty acid metabolism, amino acids, and nucleotides.** *A*, Samples sent for lipidomic analysis with SARS2E detected. *B*, Impact of SARS2E on LPCs*, C*, Carnitine and fatty acid metabolism, *D*, amino acid, and *E*, nucleotide relative amounts. Median values of n=5 HEK293T cells transfected with SARS2E with t-test values published above the respective histogram plots. *p<0.05, **p<0.01, and ***p<0.001.


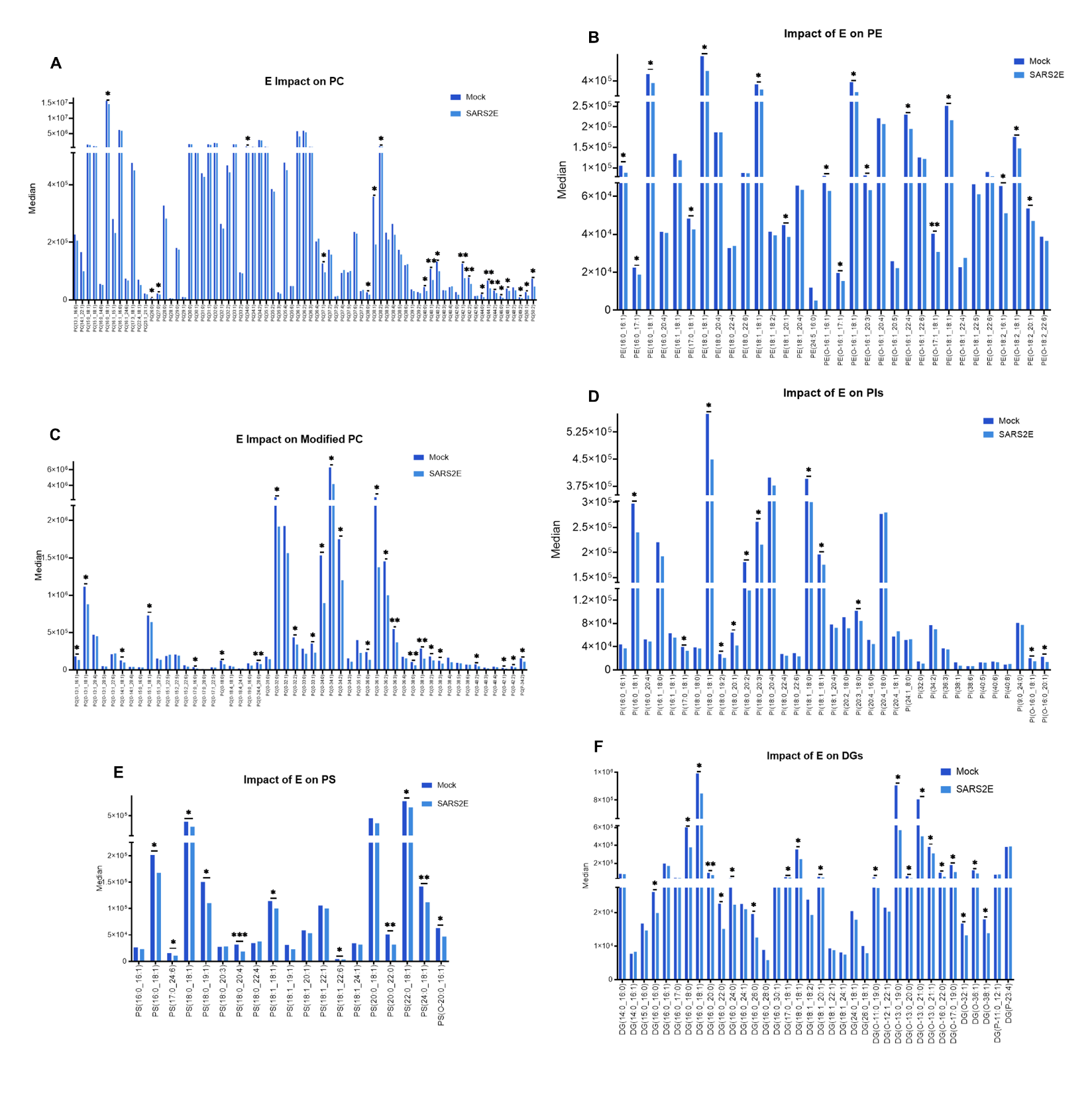


**Supplemental Figure 3. Impact of SARS2E on PCs, PE, and PIs.** *A*, Lipidomics results for PCs *B*, PEs *C*, modified PCs *D*, PIs *E*, PSs *F*, and DGs. T-test values are indicated by the respective * above the respective histogram plots. *p<0.05, **p<0.01, and ***p<0.001.

**
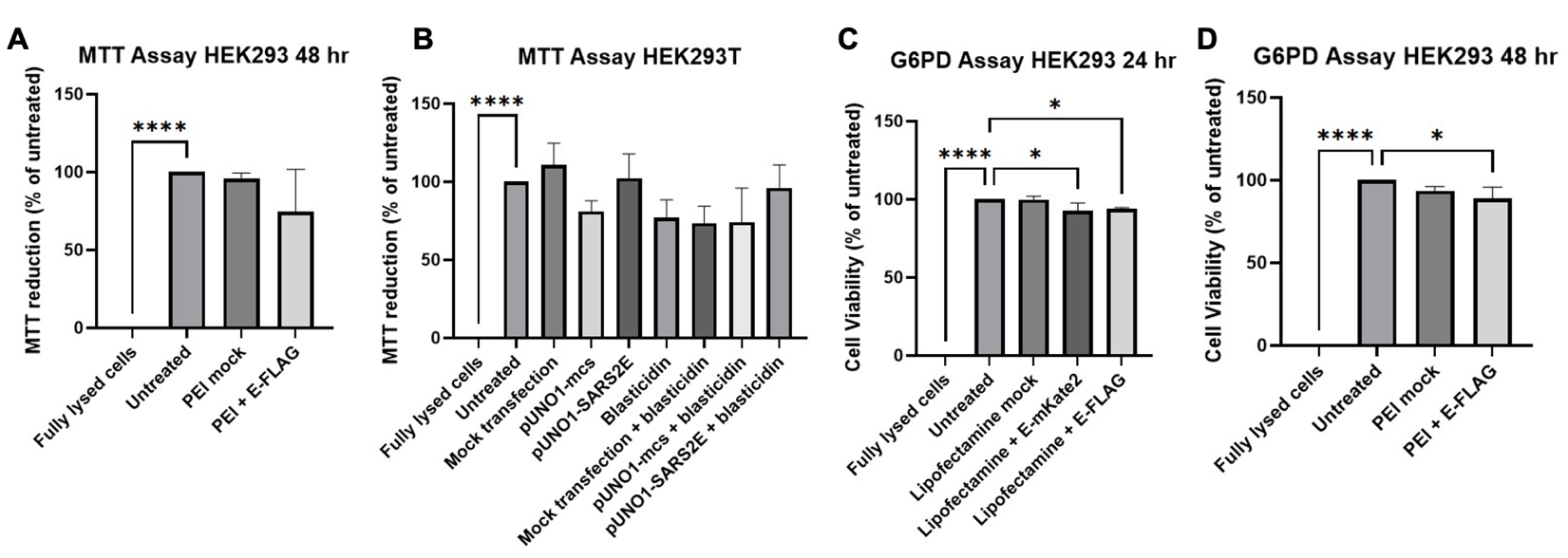
**

**Supplemental Figure 4. MTT and G6PD Cytotoxicity assays.** *A*, The MTT assay was used to assess cell proliferation for untreated HEK293 cells, lysed HEK293 cells, HEK293 cells treated with transfection reagent (PEI), or HEK293 cells transfected with PEI and the E-Flag plasmid. *B*, The MTT assay was used to assess cell proliferation for HEK293T cells untreated, lysed HEK293T cells, mock transfection with transfection reagent, Blasticidin S treatment, mock transfection with Blasticidin S treatment, or cells transfected with: pUNO-1-mcs, pUNO-1SARS2E, pUNO-1 with Blasticidin S treatment, or pUNO1-SARS2E with Blasticidin S treatment. *C*, The G6PD assay was used to quantify cytotoxicity in HEK293 cells after 24 hours for lysed cells, untreated HEK293 cells, HEK293 cells mock transfected with Lipofectamine^TM^ 2000, transfected with Lipofectamine^TM^ 2000 and E-mKate2, or transfected with Lipofectamine^TM^ 2000 and E-FLAG. *D*, The G6PD assay was used to quantify cytotoxicity in HEK293 cells after 48 hours for lysed cells, untreated cells, cells mock transfected with PEI, or cells transfected with PEI and E-FLAG. Three biological replicates were performed for all assay conditions. All samples were compared by a one-way ANOVA, where **p<0.05 and ****p<0.0001.
